# Physical Activity and Cardiovascular Risk Factors in Children from 4 to 9 Years of Age

**DOI:** 10.1186/s40798-023-00647-8

**Published:** 2023-10-24

**Authors:** Christine Delisle Nyström, Jairo H. Migueles, Pontus Henriksson, Marie Löf

**Affiliations:** 1 Group MLÖ, Department of Biosciences and Nutrition, NEO, Karolinska Institutet, 141 83 Huddinge, Sweden; 2 PROFITH “PROmoting FITness and Health Through Physical Activity” Research Group, Department of Physical Education and Sports, Faculty of Sport Sciences, University of Granada, Granada, Spain; 3 Department of Health, Medicine and Caring Sciences, Linköping University, 581 83 Linköping, Sweden

**Keywords:** Accelerometry, Cardiometabolic, Children, Vigorous physical activity

## Abstract

**Background:**

Physical activity guidelines for children encourage moderate-to-vigorous intensity activities (MVPA); however, some studies have found that only vigorous intensity activities (VPA) might promote health benefits in young children. Thus, the aim of this study is to investigate cross-sectional and 5-year longitudinal associations of VPA and MVPA with cardiovascular disease (CVD) risk factors in childhood using compositional data analysis.

**Results:**

This study utilized data from the SPINACH study (n = 411). Physical activity was measured with accelerometers at 4- and 9-years of age. CVD risk factors were measured at 9-years of age, and included blood pressure (BP), lipid metabolism, and glucose metabolism biomarkers, as well as a continuous metabolic syndrome risk score (MetS). Cross-sectional and longitudinal linear regression models were built using compositional data analysis standards. Cross-sectionally, reallocating time to VPA from lower-intensity behaviours at 9-years was associated with lower waist circumference (B = − 3.219, *P* = 0.002), diastolic BP (B = − 1.836, *P* = 0.036), triglycerides (B = − 0.214, *P* < 0.001), glucose (B = − 0.189, *P* = 0.033), insulin (B = − 2.997, *P* < 0.001), and HOMA-IR (B = − 0.778, *P* < 0.001). Similarly, reallocating time to VPA at 4-years was associated with lower MetS (B = − 0.831, *P* = 0.049), waist circumference (B = − 4.211, *P* = 0.015), systolic BP (B = − 5.572, *P* = 0.015), diastolic BP (B = − 2.931, *P* = 0.044), triglycerides (B = − 0.229, *P* = 0.034), glucose (B = − 0.325, *P* = 0.032), insulin (B = − 5.114, *P* = 0.001), and HOMA-IR (B = − 0.673, *P* = 0.001) at 9-years. Reallocations of time to MVPA at 4- or 9-years were not associated with CVD risk factors at 9-years.

**Conclusions:**

VPA was associated with CVD risk factors in children both cross-sectionally (9-years) and longitudinally (at 4- and 9-years). MVPA seemed not to be a stimulus of enough intensity to trigger these potential cardiometabolic benefits in healthy children. Thus, these findings suggest the importance of higher intensity activities, i.e., VPA already in early childhood for cardiometabolic health.

**Supplementary Information:**

The online version contains supplementary material available at 10.1186/s40798-023-00647-8.

## Key Points

- Vigorous physical activity is associated with cardiovascular disease risk factors in children both cross-sectionally and longitudinally.
- MVPA may not be a stimulus of enough intensity to trigger potential cardiometabolic benefits in healthy children.
- There is a need to promote vigorous physical activity in childhood in order to promote cardiometabolic health.

**Supplementary Information:**

The online version contains supplementary material available at 10.1186/s40798-023-00647-8.

## Background

According to the Global Matrix 4.0 from October 2022 which includes data from 57 countries, levels of physical activity (PA) of children and adolescents are still alarmingly low, with only 27–33% meeting the PA recommendation of an average of 60 min of moderate-to-vigorous PA (MVPA) per day [1]. This is concerning as MVPA has been found to be related with better physical, cognitive, and mental health outcomes in children and adolescents [2]. With regards to a specific health outcome such as cardiometabolic health, a systematic review and meta-analysis in children and adolescents found inconsistent associations between MVPA and single cardiometabolic risk factors. However, in the meta-analysis, which included seven studies, a small but statistically significant inverse association between MVPA and clustered cardiometabolic risk was observed [3]. Thus, this could be indicating that MVPA may not be of enough intensity to promote meaningful cardiometabolic health benefits in healthy children and adolescents, which has been previously suggested by Ried-Larsen et al. [4].

The World Health Organization PA and sedentary behaviour guidelines for children and adolescents between 5 and 17 years were updated in 2020 [5]. These guidelines strongly recommend aerobic activities of vigorous intensity at least 3 days per week [5]; however, no time duration was provided. A recent systematic review and meta-analysis including three prospective cohort studies in children (mean age at baseline between 7.6 and 10.2 years) found a statistically significant inverse relationship between vigorous PA (VPA) and a cardiometabolic risk score [6]. Furthermore, a few longitudinal studies in children have found stronger associations between VPA and cardiorespiratory fitness (which can be used as a proxy for cardiovascular disease (CVD) risk factors [7]) than MVPA [8, 9]. It seems that MVPA has little or no association with cardiometabolic health in children [4, 10] in apparently-healthy children; however, a key question still remains as to whether the promotion of VPA in the pre-school years can improve cardiometabolic health in later childhood.

Clearly, there is a need for more well-conducted studies investigating the associations between VPA and CVD risk factors in childhood. In the recent systematic review and meta-analysis by García-Hermoso et al. [6] they highlighted that the major limitations were that few studies have utilized compositional data analysis as well as the relatively short-follow-up duration (average 3.2 years). Compositional data analysis has been suggested to handle the interplay between the movement behaviours co-existing in the day (i.e., sleep, sedentary behaviour, and PA) and the dependency and relationships that characterize these behaviours [11–13]. Thus, the aim of this study was to investigate the cross-sectional and 5-year longitudinal associations of VPA and MVPA with CVD risk factors in early childhood using compositional data analysis.

## Methods

### Study Design and Participants

Data from three cohorts were pooled together under the umbrella of SPINACH (Studies of Prospective health determinants in Infancy and CHildhood), two birth cohorts [14, 15] and one randomized controlled trial, MINISTOP [16]. These three studies included a sample of children from the Region of Östergötland in Sweden and a coordinated follow-up measurement of PA and sociodemographic characteristics was conducted when the children from the three studies were 9 years of age. Thus, ensuring identical measures and data processing. Only the randomized controlled trial included a measurement of PA at 4 years of age; therefore, longitudinal analyses in this study only include the participants from the MINISTOP trial [16], whereas the cross-sectional analysis at 9 years of age includes participants from all three studies. To be included in the birth cohorts infants needed to be healthy singletons and born after 37 weeks of gestation [14, 15]. For the MINISTOP trial inclusion criteria consisted of a healthy 4-year-old (i.e., with no medical condition) and the baseline measurement needed to occur at 4.5 years (± 2 months) of age [16]. Children were excluded from MINISTOP if the child had a condition that would affect body size or if the parent(s) had a physical/psychological condition which would make the study too stressful for the family. Of the 632 children originally measured in all three studies, at the 9-year follow-up 411 children participated (Additional file 1: Fig. S1).

For the analyses of CVD risk factors (i.e., lipid and glycaemic metabolism biomarkers) at the 9-year follow-up participants were asked to give a blood sample and 175 children consented. The ethics committees in Linköping (2016/300-31) and Stockholm (2013/1607-31/5; 2013/2250-32; 2018/2220-32) approved this study. It was conducted in accordance with the Declaration of Helsinki and all parents provided informed consent.

### Data Collection

#### Movement Behaviours at 4 and 9 Years of Age

PA, sedentary behaviour, and sleep were continuously assessed with ActiGraph GT3X+ accelerometers (ActiGraph, Pensacola, FL, USA). The children wore them on the non-dominant wrist for 7 days (24 h/day) and data was collected using a 50 Hz sampling frequency. Participants were to wear the devices at all times, except for water-based activities. To be included in the analyses children needed to wear the accelerometer for a minimum of 16 h for at least 1 day (REF). The GGIR R package [17] was used to process the accelerometer data. In brief, the Euclidean Norm of the raw accelerations Minus One *G* with negative values rounded to zero was calculated over 5 s epochs. Periods of non-wear time were identified from the magnitude and variability of the raw accelerations measured at each accelerometer axis [18]; sleep and awake periods were identified using an automated algorithm [19, 20]; and awake time was subsequently classified into sedentary behavior (< 35 mg), or PA of light (35–199 mg), moderate (200–699 mg), or vigorous (≥ 700 mg) intensity [21, 22].

#### Cardiovascular Disease Risk Factors at 9 Years of Age

Systolic and diastolic blood pressure (BP) in mmHg were assessed with an electric sphygmomanometer (WelchAllyn, ProBP 3400 series, NY, USA) following a standard protocol (i.e., seated upright for 5 min before the measurement). BP was measured twice; however, a third measurement was conducted if the values varied by greater than 10 mmHg. The average of the measures was utilized in the analyses. Fasting venous blood samples were provided by the participants and the samples were analyzed for glucose (measured in plasma), insulin (measured in serum), total cholesterol, high density lipoprotein (HDL), low density lipoprotein (LDL) cholesterol, and triglycerides. The blood samples were analyzed at the accredited (ISO/IEC 17025) Department of Clinical Chemistry at Linköping University, Sweden. HOMA-IR was calculated as an indicator of insulin resistance as fasting insulin [μU/L] multiplied by fasting glucose [mmol/L]) divided by 22.5 [23]. Using the natural logarithm (ln) HOMA-IR values were transformed and those values were used in the statistical analyses due to the skewed distribution.

In line with the common definition of metabolic syndrome (MetS) a continuous cardiovascular risk score was calculated in accordance with previous studies [24, 25]. Therefore, the MetS score was computed as the normalized sum of sex-specific z-scores for triglycerides, inverted HDL-cholesterol (multiplied by − 1), fasting glucose, the mean of systolic and diastolic BP, and waist circumference as described previously [24, 25].

### Statistics

Descriptive characteristics of the participants were reported as mean and standard deviation (SD) or frequencies as appropriate. The analyses were performed under the principles of the compositional data analysis to account for the relative of the accelerometer-measured behaviours [11–13]. Thus, findings of this manuscript should be interpreted as the hypothetical effect of increasing VPA or MVPA while reducing the rest of the measured behaviours. Compositional descriptive statistics consisted of the geometric mean (normalized mean to the average day duration, ~ 24 h) and the variation matrix. The variation matrix summarizes the variability structure of the data by means of log-ratio variances, the lower the values the higher the inter-dependence between that pair of behaviours.

For the regression models, two different time-use compositions with either VPA or MVPA as the dominant behaviour were used (composition 1 consisted of VPA, moderate PA, light PA, sedentary behavior, and sleep; while composition 2 consisted of MVPA, light PA, sedentary behavior, and sleep). The movement behaviours were expressed as a set of isometric log-ratio (ILR) coordinates [11], which represent the effect of increasing one behaviour while decreasing the rest of the behaviours. The ILR coordinates for the movement behaviours measured at 9 years of age were fitted as explanatory variables and the CVD risk factors were fitted as outcomes. Analyses were adjusted for the child’s age and sex as well as maternal education level (i.e., university level vs. lower) and maternal body mass index (BMI) (continuous). Additionally, longitudinal models were adjusted for the change in the ILR coordinates from 4 to 9 years of age (to account for the confounding effect of the behaviours measured at two time points) and treatment group (intervention or control) [26]. Selection of covariates was done based on previous analyses in the same cohorts (REF). Daily energy intake (kJ/day) derived from a food frequency questionnaire was also tested as a confounder in the analyses and disregarded from the final models as it did not influence the associations (Additional file 1: Tables S5 and S6). Unstandardized beta-coefficients (B) for the ILR_1_ of the two compositions were examined for significance, magnitude, and direction of the relationships for the effect of increasing VPA (composition 1) or MVPA (composition 2) while proportionally reducing the rest of the behaviours in each composition. Unadjusted and adjusted estimates are provided. Plots with the predicted effect of these reallocations of time, based on the model coefficients were drawn to ease the interpretation of the findings. The results can be interpreted as demonstrating the outcome associated with reallocating time between behaviours for a hypothetical average participant in our sample.

Sensitivity analyses were conducted including only children who provided ≥ 3 valid days of accelerometer data (i.e., 18 out of 326 children at 4 years and 4 out of 398 children at 9 years of age provided less than 3 valid days); as the results remained unchanged, the data including all participants were reported. Furthermore, as the cross-sectional models include data from three different studies further sensitivity analyses were performed for the birth cohorts and the MINISTOP study separately (Additional file 1: Tables S1 and S2). The statistical software R version 4.1.2 (R Foundation for Statistical Computing) were used to conduct all analyses. Two-sided *P* values < 0.05 were considered statistically significant.

## Results

### Participant Characteristics

**Fig. 1:**
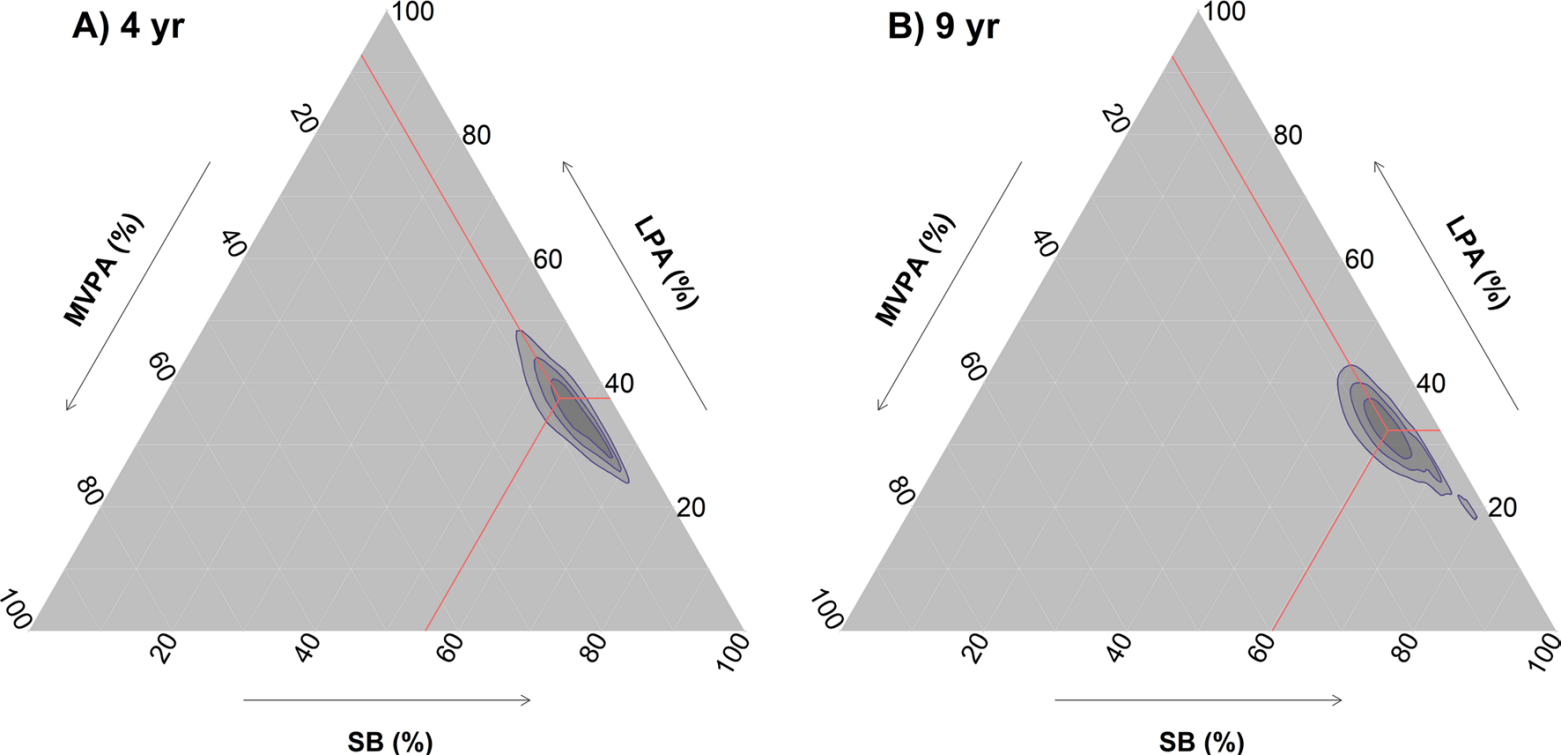
Ternary plots for the daily time-use in the movement behaviours at 4 years old (**A**) and 9 years old (**B**). The lines converge at the geometric means at 4 years old (**A**) and 9 years old (**B**). See Additional file 1: Table S3 for tabulated data on geometric means. *LPA* light physical activity, *MVPA* moderate-to-vigorous physical activity, *SB* sedentary behaviour, *VPA* vigorous physical activity

Descriptive sociodemographic characteristics and CVD risk factors of the children are presented in Table 1. Children wore the accelerometers on average for 22.4 (SD = 2.1) h/day at 4 years and 23.5 (SD = 1.0) h/day at 9 years. The movement behaviour compositions at 4 and 9 years of age are graphically presented in ternary plots in Fig. 1. The geometric mean of the movement behaviours investigated at 4 years old was 10 min of VPA, 55 min of moderate PA, 351 min of light PA, 509 min of sedentary behaviour, and 515 min of sleep per day. Correspondingly, the geometric mean at 9 years of age were 12 min of VPA, 54 min of moderate PA, 291 min of light PA, 544 min of sedentary behaviour, and 538 min of sleep per day (Additional file 1: Table S3). Participants wore the devices for an average of 22.5 (SD = 2.2) h/day at 4 years and 23.5 (SD = 1.0) h/day at 9 years; and accumulated 6.1 (SD = 1.5) valid days at 4 years and 7.0 (SD = 0.9) days at 9 years. The covariance matrices for the movement behaviours at 4 and 9 years of age are presented in Additional file 1: Table S4. At 9 years of age, the 175 children who provided blood samples were quite comparable to the 236 children that did not provide samples in terms of BMI (17.0 vs. 16.9 kg/m^2^), age (9.6 vs. 9.5 years), sex distribution (55.1% vs. 46.6% boys), maternal educational attainment (80.5% vs. 75.0% with a university degree), VPA (14.1 vs. 14.1 min/day), and MVPA (71.1 vs 70.9 min/day).

**Table 1:** Characteristics of participating children at baseline and at the 5-year follow-up

	Cross-sectional analyses		Longitudinal analyses			
			Baseline		Follow-up	
	n	Value	n	Value	n	Value
Age (year)	411	9.5 (0.1)	231	4.5 (0.1)	231	9.6 (0.1)
Height (cm)	411	139.4 (6.1)	231	107.6 (4.3)	231	139.7 (6.3)
Weight (kg)	411	33.2 (6.8)	231	18.3 (2.5)	231	33.6 (7.1)
Mother BMI (kg/m^2^)	400	24.5 (4.1)	231	24.0 (3.9)	227	24.7 (4.3)
Weight status n (%)	411		231		231	
Underweight		45 (10.9)		21 (9.1)		22 (9.5)
Normal weight		313 (76.4)		192 (83.1)		176 (76.2)
Overweight		45 (10.9)		15 (6.5)		29 (12.6)
Obesity		8 (1.3)		3 (1.3)		4 (1.7)
Mother education *n* (%)	411		231		231	
University or higher		321 (78.1)		172 (74.5)		47 (20.3)
Below university		90 (21.9)		59 (25.5)		184 (79.7)
*CVD risk factors*						
Systolic BP (mmHg)	409	111 (9)			230	111 (9)
Diastolic BP (mmHg)	409	69 (6)			230	68 (6)
Glucose (mmol/l)^a^	173	5.1 (0.4)			86	5.1 (0.3)
Insulin (mmol/l)^b^	169	7.4 (3.8)			85	7.4 (4.0)
HOMA-IR	166	1.7 (0.9)			84	1.7 (1.0)
Total cholesterol (mmol/l)^a^	175	4.2 (0.7)			87	4.2 (0.6)
LDL cholesterol (mmol/l)^a^	175	2.2 (0.6)			87	2.2 (0.6)
HDL cholesterol (mmol/l)^a^	175	1.7 (0.4)			87	1.7 (0.4)
Triglycerides (mmol/l)^a^	175	0.6 (0.2)			87	0.7 (0.3)
Waist circumference (cm)	411	64.4 (7.3)			231	65.1 (7.6)
MetS score (*z* score)	173	0.0 (1.0)			85	0.0 (1.0)

Data presented as mean (SD) unless otherwise stated

*CVD* cardiovascular disease, *BP* blood pressure, *HDL* high-density lipoprotein, *HOMA-IR* homeostatic model assessment of insulin resistance, *LDL* light-density lipoprotein, *MetS* metabolic syndrome score, *SD* standard deviation

^a^Measured in plasma

^b^Measured in serum

### Cross-Sectional Associations at 9 Years of Age

**Fig. 2:**
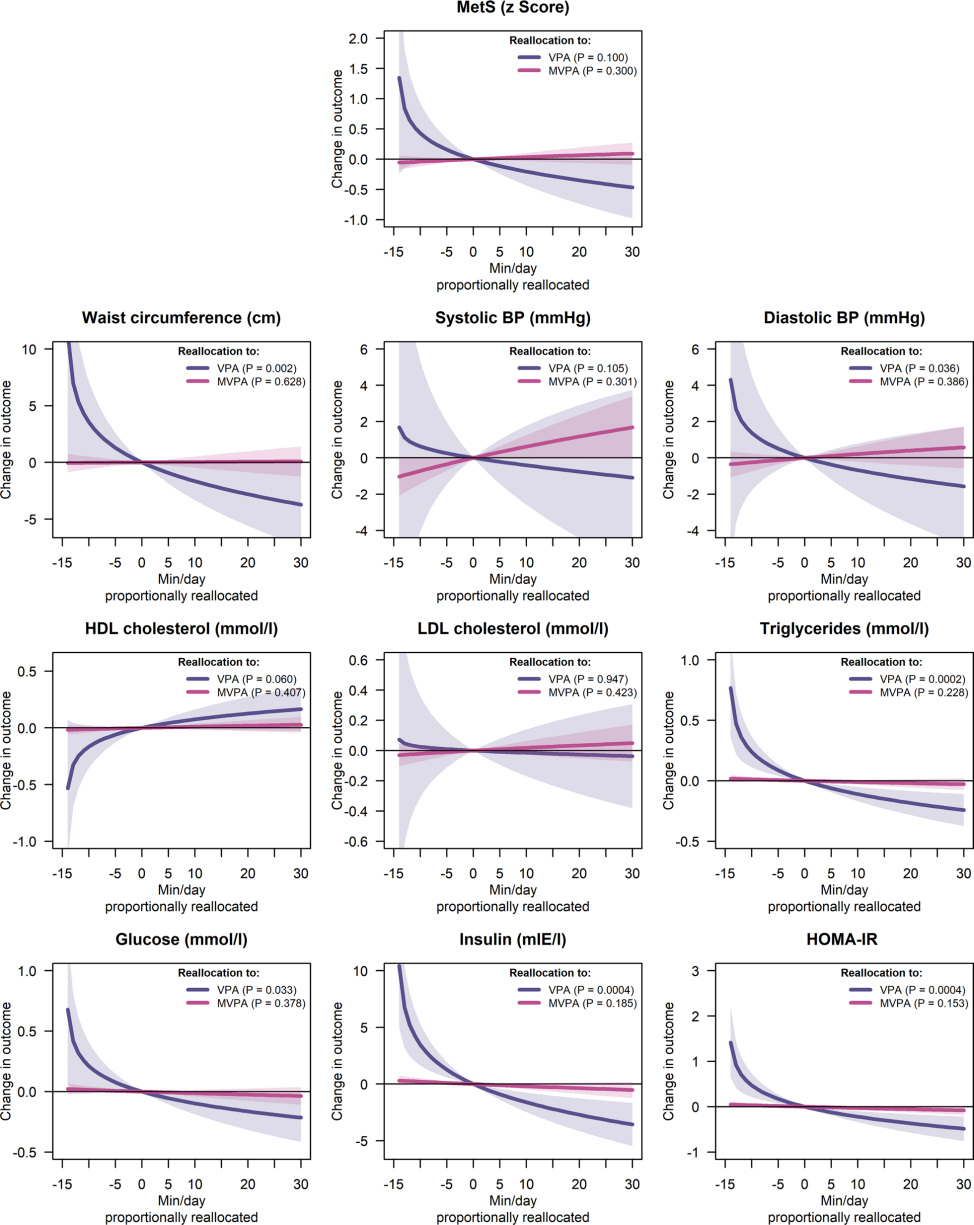
Cross-sectional associations of VPA relative to moderate PA, light PA, sedentary behaviour, and sleep (purple lines) and MVPA relative to light PA, SB, and sleep (pink lines) with CVD risk factors at 9 years of age. The lines represent the expected change in the outcomes upon increasing the dominant behavior while proportionally reducing the others. Models are adjusted for the child’s age and sex as well as maternal education level and body mass index as measured at the 9-year assessment. *BP* blood pressure, *CVD* cardiovascular disease, *HDL* high-density lipoprotein, *HOMA-IR* homeostatic model assessment of insulin resistance, *LDL* light-density lipoprotein, *MetS* metabolic syndrome score, *MVPA* moderate-to-vigorous physical activity, *PA* physical activity, *VPA* vigorous physical activity

The cross-sectional associations of VPA and MVPA relative to the rest of the behaviours with CVD risk factors measured at 9 years of age are graphically represented in Fig. 2 (tabulated data presented in Additional file 1: Table S5). Reallocating time to VPA from lower-intensity behaviours was associated with a lower waist circumference (B = − 3.219, *P* = 0.002), diastolic BP (B = − 1.836, *P* = 0.036), triglycerides (B = − 0.214, *P* < 0.001), glucose (B = − 0.189, *P* = 0.033), insulin (B = − 2.997, *P* < 0.001), and HOMA-IR (B = − 0.778, *P* < 0.001). However, increasing MVPA relative to the lower-intensity behaviours was not associated with any of the CVD risk factors at 9 years of age.

### Longitudinal Prospective Associations from 4 to 9 Years of Age

**Fig. 3:**
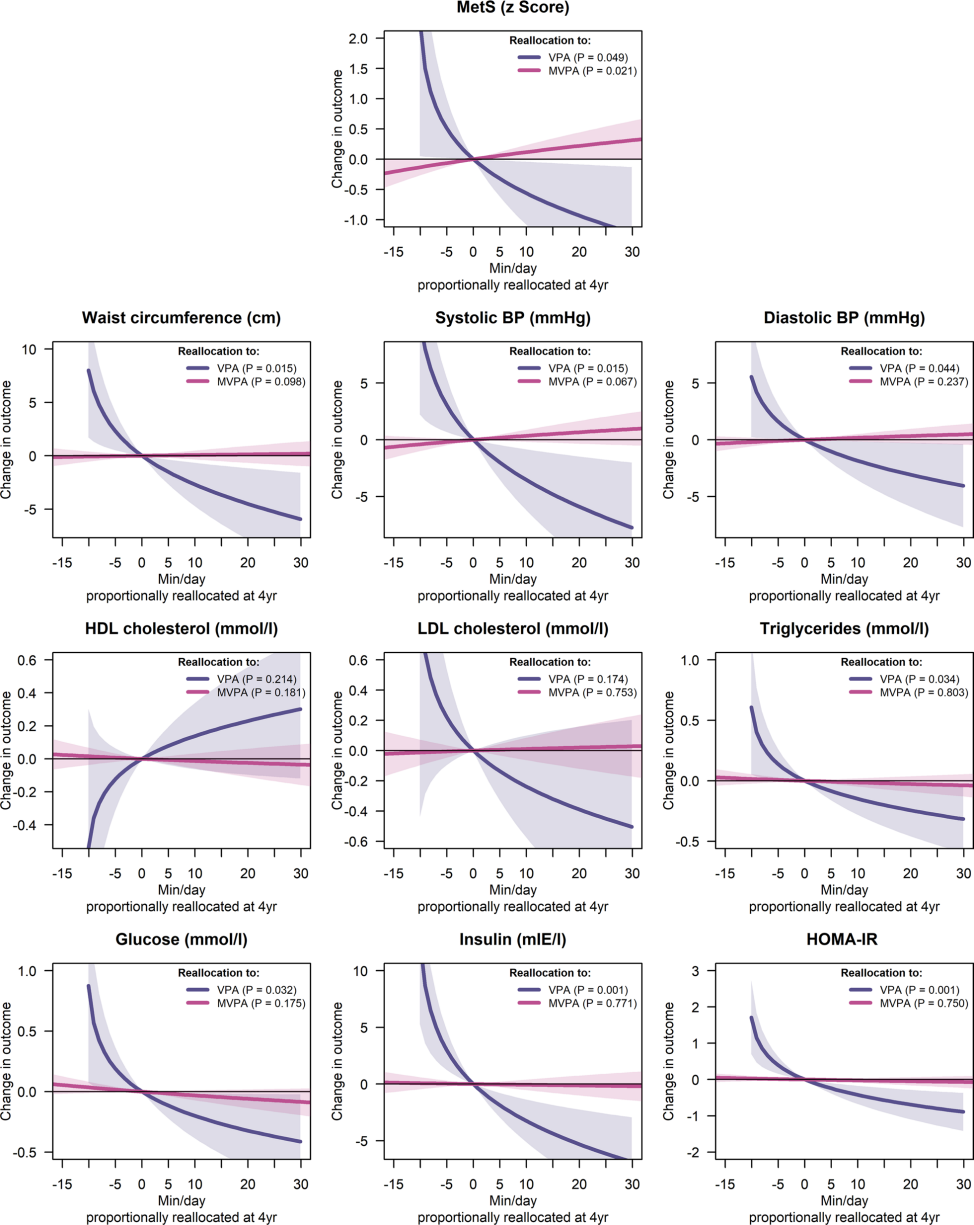
Longitudinal associations of VPA relative to moderate PA, light PA, sedentary behaviour, and sleep (purple lines) and MVPA relative to light PA, sedentary behaviour, and sleep (pink lines) at 4 years of age with CVD risk factors at 9 years of age. The lines represent the expected change in the outcomes upon increasing the dominant behavior while proportionally reducing the others. Models are adjusted for the child’s age, maternal education level and body mass index as measured at both the 4- and 9-year assessment, the change in the movement behaviours from 4 to 9 years, and treatment group (intervention or control). *BP* blood pressure, *CVD* cardiovascular disease, *HDL* high-density lipoprotein, *HOMA-IR* homeostatic model assessment of insulin resistance, *LDL* light-density lipoprotein, *MetS* metabolic syndrome score, *MVPA* moderate-to-vigorous physical activity, *PA* physical activity, *VPA* vigorous physical activity

The longitudinal associations of VPA and MVPA relative to the rest of behaviours measured at 4 years of age with CVD risk factors measured at 9 years of age are represented in Fig. 3 (tabulated data presented in Additional file 1: Table S6). Increasing the time in VPA at the expense of the other behaviours at 4 years was associated with lower values for several CVD risk factors measured at 9 years of age, i.e., MetS score (B = − 0.831, *P* = 0.049), waist circumference (B = − 4.211, *P* = 0.015), systolic BP (B = − 5.572, *P* = 0.015), diastolic BP (B = − 2.931, *P* = 0.044), triglycerides (B = − 0.229, *P* = 0.034), glucose (B = − 0.325, *P* = 0.032), insulin (B = − 5.114, *P* = 0.001), and HOMA-IR (B = − 0.673, *P* = 0.001). MVPA relative to the other behaviours at 4 years of age showed a positive association with the MetS score measured at 9 years of age (B = 1.213, *P* = 0.021).

## Discussion

To the best of our knowledge, this is the first study in childhood utilizing compositional data analysis to investigate longitudinal associations between PA and CVD risk factors in childhood. In the present study in both cross-sectional and longitudinal analyses increasing time in VPA relative to the other behaviours was statistically significantly associated with the lowering of several CVD risk factors. However, no statistically significant associations between CVD risk factors and MVPA (relative to light PA, sedentary behaviour, and sleep) in neither longitudinal nor cross-sectional analyses were found. Thus, these results are demonstrating the importance of promoting VPA for cardiometabolic health in childhood.

### Cross-Sectional Associations

Using compositional data analysis, we found that increasing MVPA in relation to lower intensity behaviours was not significantly related with CVD risk factors. This is contradictory to another cross-sectional study utilizing compositional data analysis where Carson et al. [27] found that MVPA in relation to other movement behaviours was negatively related with the log waist circumference, systolic and diastolic BP, triglycerides, and insulin. The inconsistent results observed between the studies could be due to age differences (9 years present study vs. 6–17 years Carson et al. [27]), the different activity monitors and placement used (wrist-worn ActiGraph present study compared to the waist-worn Actical Carson et al. [27]), as well as different processing techniques for accelerometer data. For example, Carson et al. [27] placed accelerometers on the hip and used cut-points based on minute-level accelerations, while this study used wrist-worn accelerometers and aggregated the accelerations over 5-s epochs. The hip and wrist placement record different movement patterns [28], which may result in different activities being captured. Furthermore, longer epochs smooth out the incidental short bouts of PA, which causes more intensity to be needed to reach the MVPA threshold [29]. As such, it might be that the MVPA defined by Carson et al. [27] is explained by activities of a different type and higher intensity than the MVPA defined in this study.

With regards to VPA, we found the reallocation of time from lower-intensity behaviours to VPA was significantly associated with a lower waist circumference, diastolic BP, triglycerides, glucose, insulin, and HOMA-IR. These results are in line with the 2003–2006 US National Health and Nutrition and Examination Survey in children aged 6–17 years utilizing compositional data analysis where they found that spending more time in VPA in relation to lower intensity behaviours was negatively related to log waist circumference and positively related to HDL cholesterol [30]. Furthermore, it is important to note that Carson et al. [30] did not include MVPA in the analyses; however, with regards to moderate PA they found no associations with cardiometabolic indicators in their adjusted models. Thus, the results from the current study coupled with the study by Carson et al. [30] are indicating that VPA may be more important than lower intensity PA with regards to cardiometabolic health in healthy children.

### Longitudinal Associations

In the current study we observed that increasing time in VPA at 4 years of age at the expense of the other behaviours was inversely associated with waist circumference, systolic BP, triglycerides, insulin, HOMA-IR, and MetS score at 9 years of age. To date, no other study has utilized compositional data analysis to investigate longitudinal associations between movement behaviours and cardiometabolic health in childhood. Using linear regression, Väistö et al. [31] in a 2-year follow-up of children with a mean age of 7.6 years at baseline found that the change in VPA was significantly negatively related with the change in waist circumference, insulin, triglycerides, HOMA-IR, and a cardiometabolic risk score as well as significantly positively associated with HDL cholesterol. Furthermore, a prospective study using linear regression analysis in 8- to 10-year-old children with a 6-year follow-up observed a significant inverse relationship between average time spent in VPA and a cardiometabolic risk score [4]. Interestingly, in the same study no association between the average time spent in MVPA and a cardiometabolic risk score was found [4], further providing evidence that VPA may be more important than MVPA for cardiometabolic health in childhood.

A statistically significant positive association between MVPA in relation to the other behaviours at 4-years of age and a MetS score measured at 9-years of age was observed in the current study. When examining the individual components of the MetS score it indicates that this positive association is mainly being driven by small-sized associations with systolic and diastolic BP. For example, increasing 30 min/day of MVPA at expenses of the lower-intensity behaviours was non-significantly associated with a change of + 0.7 mmHg in systolic BP and + 0.4 mmHg in diastolic BP. The associations with the rest of the MetS components were either null or in the expected direction (yet none of them were significant). Therefore, these associations seem not to be clinically meaningful, and likely related to randomness given the number of tests conducted.

### Strengths and Limitations

Strengths of this study include its longitudinal design, relatively long follow-up (5 years), the use of state-of-the-art data processing techniques for the accelerometer data [13, 17, 29], and the utilization of compositional data analysis. This study is limited by the small number of participants included in the longitudinal analyses which might have limited our capacity to detect low-to-moderate association sizes. However, it is important to highlight that the children who provided blood samples and included in the longitudinal analyses are representative of the entire MINISTOP sample [16] with regards to age, sex, BMI, as well as time spent in MVPA and VPA at both 4 and 9 years of age. An additional limitation is the fact that MINISTOP was a randomized controlled trial. Despite this, it is valuable to note that this multi-component intervention focusing primarily on diet took place between 4.5 and 5 years of age and no statistically significant effect was observed specifically for PA (secondary outcome) at either the 5-year [16] or 5.5-year [32] follow-ups. Nevertheless, we adjusted the models in the longitudinal analyses for treatment allocation in the MINISTOP study. Additionally, as in any observational study, there might be some residual confounding that was not considered in the analyses, e.g., diet. However, adjusting for energy intake assessed through food frequency questionnaires at 4 and 9 years of age did not change our estimates (results now shown). Finally, the educational attainment of the mothers participating in this study was slightly higher than the general Swedish population [33], which may limit the generalizability of the results. However, the participating mothers had a similar mean BMI to that of the general Swedish adult population [34].

### Implications and Future Research

Currently, children and adolescents are recommended to do at least 60 min of MVPA on average across the week, due to the strong evidence for numerous health benefits [5]. Furthermore, in the WHO PA guidelines for children and adolescents [5] it is strongly recommended to perform aerobic activities of vigorous intensity at least three times per week. In a recent systematic review by García-Hermoso et al. [6] investigating VPA and health outcomes using prospective studies in children and adolescents, they concluded that their results provide the groundwork for the need to strengthen the VPA recommendation. The results from this study provide additional support for their conclusion and provides the first longitudinal evidence using compositional data analysis showing the importance of VPA in relation to cardiometabolic health in childhood. Additionally, we observed parallel clinically meaningful associations of VPA with indicators of central fatness, blood pressure, lipid metabolism, and glycemic regulation both cross sectionally and longitudinally. However, there is a need for more prospective studies with larger sample sizes as well as well-designed randomized controlled trials to investigate to what extent VPA is more important than MVPA in children.

## Conclusion

In both cross-sectional and longitudinal analyses VPA (relative to the remaining behaviours) was found to be inversely associated with several CVD risk factors in childhood. Furthermore, there was a lack of associations observed between MVPA relative to lower-intensity behaviours and CVD risk factors in both cross-sectional and longitudinal analyses. Thus, these findings suggest the importance of the promotion of VPA in childhood for cardiometabolic health.

### Supplementary Information

**Additional file 1.** Additional tables and figures.

## Data Availability

The datasets supporting the conclusions are available upon reasonable requests to the corresponding author.

## Abbreviations

BP: Blood pressure
CVD: Cardiovascular disease
HDL: High-density lipoprotein
HOMA-IR: Homeostatic model assessment of insulin resistance
LDL: Low-density lipoprotein
MetS: Metabolic syndrome
MVPA: Moderate-to-vigorous physical activity
PA: Physical activity
SD: Standard deviation
VPA: Vigorous physical activity
