# Supplementary material for: Physical Activity and Cardiovascular Risk Factors in Children from 4 to 9 Years of Age
Source: Sports Med Open. 2023 Oct 24;9:99. doi: 10.1186/s40798-023-00647-8 (PMC10597983; doi:10.1186/s40798-023-00647-8)
Supplement: Supplementary file 1 — Additional file 1. Additional tables and figures. [file 40798_2023_647_MOESM1_ESM.docx]

**Table S1.** Cross-sectional associations of movement behaviours at 4 years with CVD risk factors at 9 years of age separated by study cohort, unadjusted models.

| **Dominant behavior** | **Outcome** | **Birth cohorts** | | |  | **MINISTOP** | | |  |
| --- | --- | --- | --- | --- | --- | --- | --- | --- | --- |
|  |  | **n** | **B** | ***P*** |  | **n** | **B** | ***P*** | |
| VPA | MetS (z score) ^a^ | 85 | -0.835 | **0.011** |  | 85 | -0.451 | 0.179 | |
| MVPA | MetS (z score) | 85 | -0.249 | 0.423 |  | 85 | 0.387 | 0.333 | |
| VPA | Systolic BP (mmHg) | 173 | -2.858 | 0.053 |  | 224 | -2.855 | 0.099 | |
| MVPA | Systolic BP (mmHg) | 173 | -1.287 | 0.385 |  | 224 | 1.57 | 0.412 | |
| VPA | Diastolic BP (mmHg) | 173 | -4.389 | 0.064 |  | 224 | -2.213 | **0.042** | |
| MVPA | Diastolic BP (mmHg) | 173 | -2.268 | 0.341 |  | 224 | 0.594 | 0.623 | |
| VPA | HDL cholesterol (mmol/l) | 86 | 0.058 | 0.589 |  | 86 | 0.253 | **0.034** | |
| MVPA | HDL cholesterol (mmol/l) | 86 | 0.123 | 0.225 |  | 86 | 0.067 | 0.641 | |
| VPA | LDL cholesterol (mmol/l) | 86 | 0.014 | 0.949 |  | 86 | -0.208 | 0.267 | |
| MVPA | LDL cholesterol (mmol/l) | 86 | -0.112 | 0.596 |  | 86 | -0.13 | 0.562 | |
| VPA | Triglycerides (mmol/l) | 86 | -0.115 | 0.111 |  | 86 | -0.34 | **<0.001** | |
| MVPA | Triglycerides (mmol/l) | 86 | -0.084 | 0.215 |  | 86 | -0.126 | 0.233 | |
| VPA | Glucose (mmol/l) ^b^ | 85 | -0.140 | 0.293 |  | 85 | -0.174 | 0.111 | |
| MVPA | Glucose (mmol/l) ^b^ | 85 | 0.078 | 0.534 |  | 85 | -0.084 | 0.516 | |
| VPA | Insulin (mIE/l) ^c^ | 82 | -3.055 | **0.012** |  | 84 | -4.394 | **<0.001** | |
| MVPA | Insulin (mIE/l) ^c^ | 82 | -2.541 | **0.031** |  | 84 | -1.878 | 0.219 | |
| VPA | HOMA-IR | 80 | -0.479 | **0.010** |  | 83 | -0.552 | **0.001** | |
| MVPA | HOMA-IR | 80 | -0.350 | **0.049** |  | 83 | -0.323 | 0.100 | |
| VPA | Waist circumference (cm) | 174 | -2.045 | 0.212 |  | 224 | -5.21 | **<0.001** | |
| MVPA | WC circumference (cm) | 174 | -0.454 | 0.784 |  | 224 | -0.15 | 0.925 | |

Abbreviations: BP: blood pressure, CVD: cardiovascular disease, HDL: high-density lipoprotein, HOMA-IR: Homeostatic Model Assessment of Insulin Resistance, LDL: light-density lipoprotein, MetS: metabolic syndrome score, MVPA: moderate-to-vigorous physical activity, VPA: vigorous physical activity.

^a^ MetS score calculated using the normalized sum of sex-specific z scores for triglycerides, inverted HDL cholesterol, fasting glucose, and the average of systolic and diastolic BP.

^b^ Measured in plasma.

^c^ Measured in serum.

**Table S2.** Cross-sectional associations of movement behaviours at 4 years with CVD risk factors at 9 years of age separated by study cohort, adjusted models.

| **Dominant behavior** | **Outcome** | **Birth cohorts** | | |  | **MINISTOP** | | |  |
| --- | --- | --- | --- | --- | --- | --- | --- | --- | --- |
|  |  | **n** | **B** | ***P*** |  | **n** | **B** | ***P*** | |
| VPA | MetS (z score) ^a^ | 82 | -0.498 | 0.134 |  | 83 | -0.236 | 0.456 | |
| MVPA | MetS (z score) | 82 | -0.010 | 0.977 |  | 83 | 0.831 | 0.089 | |
| VPA | Systolic BP (mmHg) | 167 | -1.782 | 0.247 |  | 220 | -2.35 | 0.179 | |
| MVPA | Systolic BP (mmHg) | 167 | 0.196 | 0.909 |  | 220 | 2.379 | 0.290 | |
| VPA | Diastolic BP (mmHg) | 167 | -1.952 | 0.428 |  | 220 | -1.891 | 0.081 | |
| MVPA | Diastolic BP (mmHg) | 167 | 1.078 | 0.696 |  | 220 | 1.318 | 0.345 | |
| VPA | HDL cholesterol (mmol/l) | 83 | 0.060 | 0.611 |  | 84 | 0.214 | 0.080 | |
| MVPA | HDL cholesterol (mmol/l) | 83 | 0.111 | 0.378 |  | 84 | -0.059 | 0.753 | |
| VPA | LDL cholesterol (mmol/l) | 83 | 0.232 | 0.345 |  | 84 | -0.154 | 0.424 | |
| MVPA | LDL cholesterol (mmol/l) | 83 | 0.194 | 0.457 |  | 84 | 0.089 | 0.764 | |
| VPA | Triglycerides (mmol/l) | 83 | -0.079 | 0.309 |  | 84 | -0.33 | **<0.001** | |
| MVPA | Triglycerides (mmol/l) | 83 | -0.042 | 0.612 |  | 84 | -0.122 | 0.371 | |
| VPA | Glucose (mmol/l) ^b^ | 82 | -0.228 | 0.117 |  | 83 | -0.162 | 0.142 | |
| MVPA | Glucose (mmol/l) ^b^ | 82 | -0.037 | 0.811 |  | 83 | -0.265 | 0.115 | |
| VPA | Insulin (mIE/l) ^c^ | 79 | -2.079 | 0.116 |  | 82 | -3.957 | **0.001** | |
| MVPA | Insulin (mIE/l) ^c^ | 79 | -1.875 | 0.191 |  | 82 | -0.657 | 0.725 | |
| VPA | HOMA-IR | 77 | -0.364 | 0.074 |  | 81 | -0.494 | **0.001** | |
| MVPA | HOMA-IR | 77 | -0.239 | 0.280 |  | 81 | -0.205 | 0.401 | |
| VPA | Waist circumference (cm) | 168 | -1.291 | 0.421 |  | 220 | -4.412 | **0.001** | |
| MVPA | WC circumference (cm) | 168 | -1.303 | 0.471 |  | 220 | 0.057 | 0.974 | |

Abbreviations: BP: blood pressure, CVD: cardiovascular disease, HDL: high-density lipoprotein, HOMA-IR: Homeostatic Model Assessment of Insulin Resistance, LDL: light-density lipoprotein, MetS: metabolic syndrome score, MVPA: moderate-to-vigorous physical activity, VPA: vigorous physical activity.

Adjusted for the child’s allocation group, age and sex, maternal education level and body mass index as measured at both the 4 year and the 9year assessment, and the change in movement behaviours from 4 to 9 years.

^a^ MetS score calculated using the normalized sum of sex-specific z scores for triglycerides, inverted HDL cholesterol, fasting glucose, and the average of systolic and diastolic BP.

^b^ Measured in plasma.

^c^ Measured in serum.

**Table S3.** Geometric mean for the daily time-use in movement behaviours.

|  |  | 4 years (min/day) | 9 years (min/day) |
| --- | --- | --- | --- |
| Vigorous |  | 10.1 | 11.9 |
| Moderate |  | 54.6 | 53.9 |
| Light |  | 351.0 | 291.4 |
| Sedentary |  | 509.3 | 544.5 |
| Sleep |  | 515.0 | 538.3 |

**Table S4.** Covariance matrices for the daily time-use in movement behaviors.

|  |  | Vigorous | Moderate | Light | Sedentary | Sleep |
| --- | --- | --- | --- | --- | --- | --- |
| *Baseline (4 years)* |  |  |  |  |  |  |
| Vigorous |  |  | 0.21 | 0.32 | 0.45 | 0.34 |
| Moderate |  | 0.20 |  | 0.13 | 0.39 | 0.20 |
| Light |  | 0.32 | 0.13 |  | 0.19 | 0.09 |
| Sedentary |  | 0.45 | 0.39 | 0.19 |  | 0.16 |
| Sleep |  | 0.34 | 0.20 | 0.09 | 0.16 |  |
|  |  | Vigorous | Moderate | Light | Sedentary | Sleep |
| *Follow-up (9 years)* |  |  |  |  |  |  |
| Vigorous |  |  | 0.17 | 0.38 | 0.49 | 0.41 |
| Moderate |  | 0.17 |  | 0.12 | 0.24 | 0.17 |
| Light |  | 0.38 | 0.12 |  | 0.09 | 0.05 |
| Sedentary |  | 0.49 | 0.24 | 0.09 |  | 0.04 |
| Sleep |  | 0.41 | 0.17 | 0.05 | 0.04 |  |

Note: values close to 0 represent high covariance (dependence) between the variables.

**Table S5.** Cross-sectional associations of movement behaviours with CVD risk factors at 9 years of age.

| **Dominant behavior** | **Outcome** | **Unadjusted** | | |  | **Adjusted model** ^a^ | | |  | **Adjusted model** ^b^ | | |
| --- | --- | --- | --- | --- | --- | --- | --- | --- | --- | --- | --- | --- |
|  |  | **n** | **B** | ***P*** |  | **n** | **B** | ***P*** |  | **n** | **B** | ***P*** |
| VPA | MetS (z score) ^c^ | 170 | -0.630 | **0.007** |  | 165 | -0.369 | 0.100 |  | 159 | -0.345 | 0.121 |
| MVPA | MetS (z score) | 170 | 0.034 | 0.890 |  | 165 | 0.291 | 0.300 |  | 159 | 0.134 | 0.645 |
| VPA | Systolic BP (mmHg) | 397 | -3.316 | **0.017** |  | 387 | -2.283 | 0.105 |  | 374 | -1.899 | 0.180 |
| MVPA | Systolic BP (mmHg) | 397 | -0.087 | 0.953 |  | 387 | 1.781 | 0.301 |  | 374 | 1.208 | 0.508 |
| VPA | Diastolic BP (mmHg) | 397 | -2.428 | **0.005** |  | 387 | -1.836 | **0.036** |  | 374 | -1.653 | 0.061 |
| MVPA | Diastolic BP (mmHg) | 397 | -0.179 | 0.848 |  | 387 | 0.929 | 0.386 |  | 374 | 0.152 | 0.893 |
| VPA | HDL cholesterol (mmol/l) | 172 | 0.176 | **0.030** |  | 167 | 0.159 | 0.060 |  | 161 | 0.167 | 0.051 |
| MVPA | HDL cholesterol (mmol/l) | 172 | 0.124 | 0.150 |  | 167 | 0.088 | 0.407 |  | 161 | 0.064 | 0.569 |
| VPA | LDL cholesterol (mmol/l) | 172 | -0.132 | 0.358 |  | 167 | -0.010 | 0.947 |  | 161 | -0.010 | 0.947 |
| MVPA | LDL cholesterol (mmol/l) | 172 | -0.122 | 0.417 |  | 167 | 0.150 | 0.423 |  | 161 | 0.121 | 0.546 |
| VPA | Triglycerides (mmol/l) | 172 | -0.226 | **<0.001** |  | 167 | -0.214 | **<0.001** |  | 161 | -0.220 | **<0.001** |
| MVPA | Triglycerides (mmol/l) | 172 | -0.101 | 0.096 |  | 167 | -0.089 | 0.228 |  | 161 | -0.065 | 0.409 |
| VPA | Glucose (mmol/l) ^d^ | 170 | -0.153 | 0.067 |  | 165 | -0.189 | **0.033** |  | 159 | -0.179 | **0.044** |
| MVPA | Glucose (mmol/l) ^d^ | 170 | 0.023 | 0.794 |  | 165 | -0.097 | 0.378 |  | 159 | -0.149 | 0.201 |
| VPA | Insulin (mIE/l) ^e^ | 166 | -3.773 | **<0.001** |  | 161 | -2.997 | **<0.001** |  | 157 | -3.065 | **<0.001** |
| MVPA | Insulin (mIE/l) ^e^ | 166 | -2.246 | **0.016** |  | 161 | -1.446 | 0.185 |  | 157 | -1.520 | 0.182 |
| VPA | HOMA-IR | 163 | -0.942 | **<0.001** |  | 158 | -0.778 | **<0.001** |  | 154 | -0.788 | **<0.001** |
| MVPA | HOMA-IR | 163 | -0.478 | **0.036** |  | 158 | -0.347 | 0.198 |  | 154 | -0.396 | 0.159 |
| VPA | Waist circumference (cm) | 398 | -3.950 | **<0.001** |  | 388 | -3.219 | **0.002** |  | 375 | -3.014 | **0.003** |
| MVPA | Waist circumference (cm) | 398 | -0.116 | 0.920 |  | 388 | -0.611 | 0.628 |  | 375 | -0.655 | 0.623 |

Abbreviations: BP: blood pressure, CVD: cardiovascular disease, HDL: high-density lipoprotein, HOMA-IR: Homeostatic Model Assessment of Insulin Resistance, LDL: light-density lipoprotein, MetS: metabolic syndrome score, MVPA: moderate-to-vigorous physical activity, VPA: vigorous physical activity.

^a^ Adjusted for child’s age and sex, maternal education level, maternal body mass index as measured at the 9 year assessment.

^b^ Additionally adjusted for energy intake.

^c^ MetS score calculated using the normalized sum of sex-specific z scores for triglycerides, inverted HDL cholesterol, fasting glucose, and the average of systolic and diastolic BP.

^d^ Measured in plasma.

^e^ Measured in serum.

**Table S6.** Longitudinal associations of movement behaviours at 4 years with CVD risk factors at 9 years of age.

| **Dominant behavior** | **Outcome** | **Unadjusted** | | |  | **Adjusted model** ^a^ | | |  | **Adjusted model** ^b^ | | |
| --- | --- | --- | --- | --- | --- | --- | --- | --- | --- | --- | --- | --- |
|  |  | **n** | **B** | ***P*** |  | **n** | **B** | ***P*** |  | **n** | **B** | ***P*** |
| VPA | MetS (z score) ^c^ | 85 | -0.964 | **0.028** |  | 83 | -0.831 | **0.049** |  | 81 | -0.900 | **0.042** |
| MVPA | MetS (z score) | 85 | 0.656 | 0.154 |  | 83 | 1.213 | **0.021** |  | 81 | 1.136 | **0.044** |
| VPA | Systolic BP (mmHg) | 219 | -5.609 | **0.013** |  | 216 | -5.572 | **0.015** |  | 211 | -5.834 | **0.011** |
| MVPA | Systolic BP (mmHg) | 219 | 3.382 | 0.161 |  | 216 | 4.947 | 0.067 |  | 211 | 5.077 | 0.074 |
| VPA | Diastolic BP (mmHg) | 219 | -3.193 | **0.027** |  | 216 | -2.931 | **0.044** |  | 211 | -2.903 | **0.050** |
| MVPA | Diastolic BP (mmHg) | 219 | 0.609 | 0.691 |  | 216 | 2.019 | 0.237 |  | 211 | 1.487 | 0.412 |
| VPA | HDL cholesterol (mmol/l) | 86 | 0.235 | 0.128 |  | 84 | 0.201 | 0.214 |  | 82 | 0.241 | 0.149 |
| MVPA | HDL cholesterol (mmol/l) | 86 | -0.104 | 0.516 |  | 84 | -0.260 | 0.181 |  | 82 | -0.224 | 0.278 |
| VPA | LDL cholesterol (mmol/l) | 86 | -0.467 | 0.057 |  | 84 | -0.369 | 0.174 |  | 82 | -0.385 | 0.178 |
| MVPA | LDL cholesterol (mmol/l) | 86 | -0.064 | 0.803 |  | 84 | 0.102 | 0.753 |  | 82 | 0.096 | 0.784 |
| VPA | Triglycerides (mmol/l) | 86 | -0.289 | **0.006** |  | 84 | -0.229 | **0.034** |  | 82 | -0.203 | 0.068 |
| MVPA | Triglycerides (mmol/l) | 86 | 0.042 | 0.712 |  | 84 | 0.033 | 0.803 |  | 82 | 0.100 | 0.484 |
| VPA | Glucose (mmol/l) ^d^ | 85 | -0.274 | 0.061 |  | 83 | -0.325 | **0.032** |  | 81 | -0.308 | **0.045** |
| MVPA | Glucose (mmol/l) ^d^ | 85 | -0.115 | 0.442 |  | 83 | -0.245 | 0.175 |  | 81 | -0.163 | 0.386 |
| VPA | Insulin (mIE/l) ^e^ | 84 | -5.411 | **0.001** |  | 82 | -5.114 | **0.001** |  | 80 | -5.404 | **0.001** |
| MVPA | Insulin (mIE/l) ^e^ | 84 | -1.187 | 0.493 |  | 82 | 0.572 | 0.771 |  | 80 | 0.583 | 0.783 |
| VPA | HOMA-IR | 83 | -0.684 | **0.001** |  | 81 | -0.673 | **0.001** |  | 79 | -0.685 | **0.001** |
| MVPA | HOMA-IR | 83 | -0.252 | 0.257 |  | 81 | -0.082 | 0.750 |  | 79 | -0.021 | 0.940 |
| VPA | Waist circumference (cm) | 219 | -4.961 | **0.006** |  | 216 | -4.211 | **0.015** |  | 211 | -4.188 | **0.017** |
| MVPA | Waist circumference (cm) | 219 | 1.978 | 0.309 |  | 216 | 3.410 | 0.098 |  | 211 | 3.126 | 0.153 |

Abbreviations: BP: blood pressure, CVD: cardiovascular disease, HDL: high-density lipoprotein, HOMA-IR: Homeostatic Model Assessment of Insulin Resistance, LDL: light-density lipoprotein, MetS: metabolic syndrome score, MVPA: moderate-to-vigorous physical activity, VPA: vigorous physical activity.

^a^ Adjusted for the child’s allocation group, age and sex, maternal education level and body mass index as measured at both the 4 year and the 9year assessment**,** the change in movement behaviours from 4 to 9 years, and treatment group (intervention or control).

^b^ Additionally adjusted for energy intake at 4 and 9 years of age.

^c^ MetS score calculated using the normalized sum of sex-specific z scores for triglycerides, inverted HDL cholesterol, fasting glucose, and the average of systolic and diastolic BP.

^d^ Measured in plasma.

^e^ Measured in serum.


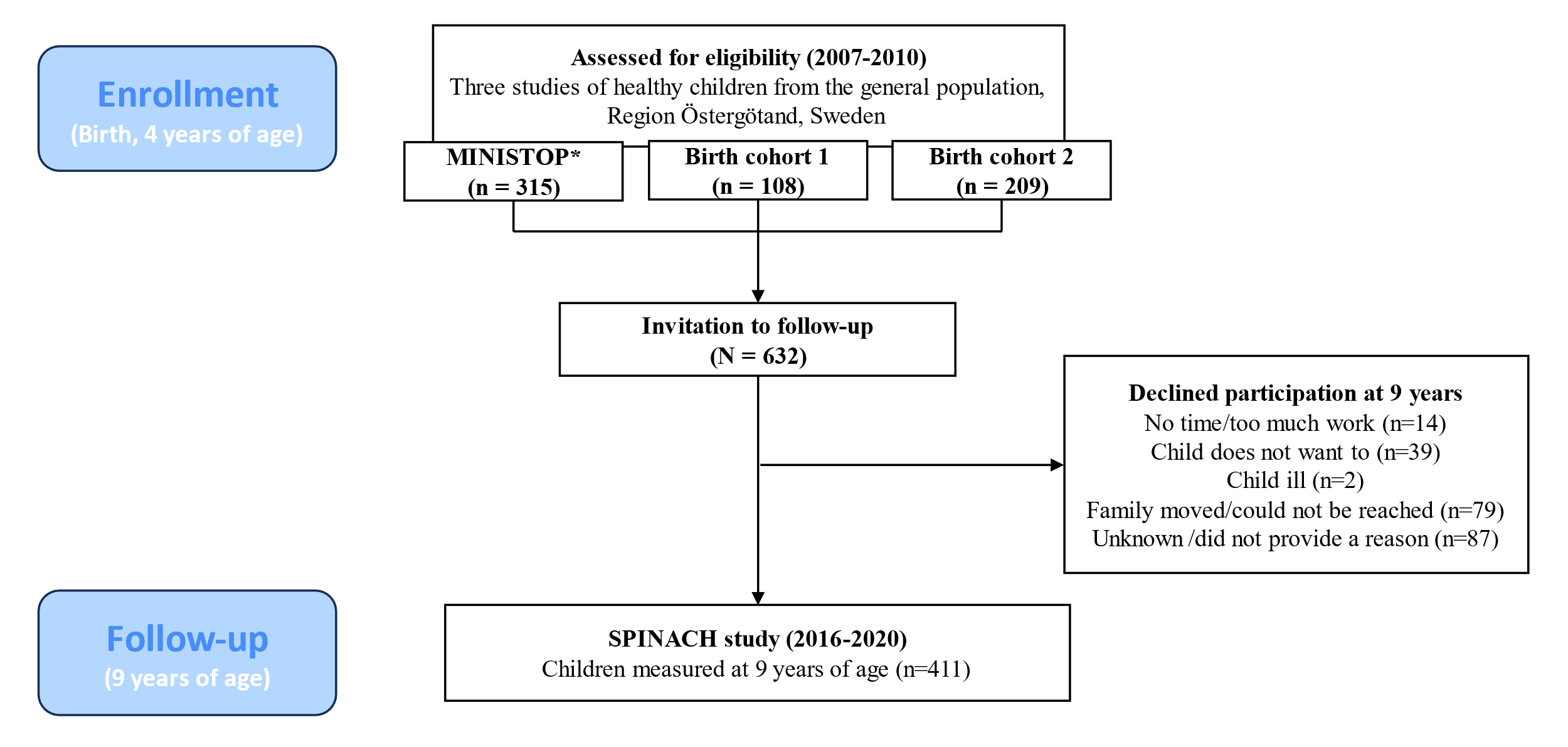


**Figure S1.** Flow diagram with the participants included in SPINACH.
